# Damiana

**Published:** 1876-06

**Authors:** 


Damiana.—Wm. B. Hazard, M.D., (Bellevue) in an interesting paper upon the treatment of the curable forms of impotency, clearly shows that damiana possesses properties far superior to any other known remedy. He reports several cases in which this agent has been administered with the most favorable results.—*St. Louis Clinical Record.*

